# An evolutionary perspective on increasing net benefits to crops from symbiotic microbes

**DOI:** 10.1111/eva.13384

**Published:** 2022-04-26

**Authors:** R. Ford Denison, Katherine E. Muller

**Affiliations:** ^1^ Ecology, Evolution, & Behavior University of Minnesota Saint Paul Minnesota USA

**Keywords:** agriculture, manipulation, mutualism, nitrogen fixation, phytohormones, rhizobia

## Abstract

Plant‐imposed, fitness‐reducing sanctions against less‐beneficial symbionts have been documented for rhizobia, mycorrhizal fungi, and fig wasps. Although most of our examples are for rhizobia, we argue that the evolutionary persistence of mutualism in any symbiosis would require such sanctions, if there are multiple symbiont genotypes per host plant. We therefore discuss methods that could be used to develop and assess crops with stricter sanctions. These include methods to screen strains for greater mutualism as resources to identify crop genotypes that impose stronger selection for mutualism. Single‐strain experiments that measure costs as well as benefits have shown that diversion of resources by rhizobia can reduce nitrogen‐fixation efficiency (N per C) and that some legumes can increase this efficiency by manipulating their symbionts. Plants in the field always host multiple strains with possible synergistic interactions, so benefits from different strains might best be compared by regressing plant growth or yield on each strain's abundance in a mixture. However, results from this approach have not yet been published. To measure legacy effects of stronger sanctions on future crops, single‐genotype test crops could be planted in a field that recently had replicated plots with different genotypes of the sanction‐imposing crop. Enhancing agricultural benefits from symbiosis may require accepting tradeoffs that constrained past natural selection, including tradeoffs between current and future benefits.

## 1 INTRODUCTION

This essay focuses on increasing benefits to crops from closely associated, that is, symbiotic, microbes. Our central hypothesis is that interactions with crop plants can have strong effects on the relative fitness of symbiotic strains that differ in mutualism. If so, then it may be possible to develop crops that impose stronger selection on microbial populations for greater mutualism. If this stronger selection increased, the relative abundance of highly beneficial strains in the pool of potential symbionts available to future crops in the same field, which could increase crop yields without requiring additional inputs.

Testing this hypothesis for a given microbial species presents three challenges. First, to test whether differences in mutualism among strains are large enough to make crop‐imposed selection worthwhile, we need reliable methods to compare benefits and costs to crops from different strains. Second, to test whether interactions with crops could strongly favor more‐beneficial strains, we need methods to measure the relative fitness of microbial strains differing in mutualism, as they interact with different crop cultivars. Finally, we need methods to assess the impact of crop‐imposed selection for greater microbial mutualism in its agricultural context. This is because increases in microbial benefits to crops would need to be substantial to be included among the many issues plant breeders and farmers must consider in choosing cultivars. The three sections that follow address these three challenges. We focus on how crops affect the evolution of microbes, rather than on coevolution, because most crop production now uses purchased seed, usually produced at distant locations.

## 2 COMPARING BENEFITS AND COSTS TO CROP PLANTS FROM DIFFERENT SYMBIONT STRAINS

There are at least two reasons that it might be worthwhile to compare benefits to crop plants from different strains of a given microbial species. First, although it is often difficult to displace existing symbiont strains, as discussed below, inoculating crops with more‐beneficial strains may occasionally be useful. In such cases, comparisons among strains are needed to pick the best inoculants. Second, and more commonly, plant‐imposed selection may be needed for more‐beneficial strains to establish and persist. In that case, a panel of strains differing in benefits to crops will be a useful or essential resource for comparing the ability of different crop genotypes to impose that selection.

After briefly considering inoculation, we first assess the challenges in comparing benefits and costs of different strains even in the simplest case where they provide a single, measurable benefit, exemplified by nitrogen fixation. For example, we discuss how measuring the carbon cost to the plant of supporting nitrogen‐fixing root nodules can explain why a rhizobia strain that diverts more resources to its own use is less‐beneficial to its plant host.

We then consider strains whose effects involve multiple benefits or context‐dependent trade‐offs (e.g., hormonal manipulation of water‐use by plants). To compare net effects (benefits minus costs) of different strains on plant growth, we argue that mixing strains in two or more ratios may be more reliable than single‐strain inoculation.

### 2.1 When might inoculation be beneficial?

Inoculation with more‐beneficial symbionts often fails to displace less‐beneficial strains in bulk soil. For example, field tests with seven legume crops found that inoculation with nitrogen‐fixing rhizobia bacteria only increased yields reliably when there were fewer than 10 competing rhizobia per gram of soil (Thies et al., 1991). That is orders of magnitude less than the typical rhizobia population size for soils where a given legume species has grown previously. The problem is not necessarily that inoculum strains are intrinsically less competitive, although introduced strains may be poorly adapted to local conditions. Instead, limited mobility of seed‐applied rhizobia limits their abundance (relative to established strains) deeper in the soil. This problem has been recognized for at least 30 years (McDermott & Graham, 1989) but improved inoculation methods may be possible (Iturralde et al., 2019).

Meanwhile, inoculation might be beneficial in a few circumstances where competition with less‐beneficial strains is not an issue. One such circumstance is when strains introduced by inoculation significantly outnumber resident strains. This may sometimes occur if soil conditions limit symbiont population sizes. For example, inoculating soybeans with soybean‐nodulating rhizobia is predicted to be more beneficial for fields with high or low pH or sandy soil (Abendroth et al., 2006).

Another scenario where inoculation is likely to be beneficial is when benefits are localized and short‐term. One example would be a microbial inoculum that is applied to seeds and whose main function is to improve germination and early growth, for example, by preventing colonization by a seed pathogen (Khan et al., 2006). Phosphorous solubilization around seeds (Rengel & Marschner, 2005) would not require establishment of inoculum strains in the bulk soil and could be particularly useful as plant breeders decrease seed content of the indigestible, polluting form of phosphorus, phytate (Yamaji et al., 2017). However, microbial solubilization of phosphorus is not a long‐term replacement for external inputs, as nutrients exported in harvested crops need to be replaced. Similarly, a microbe that hormonally manipulates plant phenotypes, analogous to hormonal growth regulators used for fruit thinning in orchards (Petracek et al., 2003), might only be needed for a short time. In fact, persistence of strains whose effects are only beneficial under some circumstances could pose unintended trade‐offs for future crops.

### 2.2 Measuring nutrient contributions from microbes: isotope methods

Measuring benefits and costs to host plants may be easier for nutrient‐for‐carbon exchange mutualisms than for defense against pathogens, for example, because it is easier to automate chemistry than biology. But measuring resource exchanges can still pose challenges. Isotope methods can be used to quantify P and N supply to plants from mycorrhizae, or N supply to plants from symbiotic N fixation. Quantifying N and P from mycorrhizae requires supplying an isotope‐enriched nutrient source that can be accessed by fungal hyphae, but not by plant roots, usually by enclosing in a fine mesh (Thirkell et al., 2020). Such approaches are useful for pot experiments, but may not be feasible for quantifying mycorrhizal P and N supply in a field setting.

Isotope methods can be used to quantify the amount of nitrogen that has been fixed, even in the field (Unkovich et al., 2008). The natural‐abundance technique takes advantage of the fact that soil N typically contains a higher abundance of the 15N stable isotope than fixed N does (Shearer & Kohl, 1986). However, natural‐abundance estimates are sensitive to measurement error due to low levels of naturally occurring 15N, and uncertainty in the parameters used to estimate symbiotic N fixation (Chalk & Craswell, 2018; Unkovich et al., 1994). Adding a 15N‐enriched soil amendment can reduce measurement error by raising the 15N levels in soil (Chalk, 1985). However, this method may be sensitive to variation in plant rooting depth and developmental timing, as it is difficult to achieve a uniform distribution of 15N throughout the soil profile and over time (Unkovich et al., 2008). Use of 15N amendments may also limit future use of natural‐abundance methods at the same site. In either case, accuracy depends on how closely nonfixing reference plants match the legume in traits such as soil N uptake.

### 2.3 Measuring cumulative nitrogen fixed versus nitrogen‐fixation rate

Measuring total N content of a legume grown with atmospheric N as its only external N source is simpler and less expensive than methods based on isotopes. However, the N contribution from the seed may be a source of error, depending on how early the plant is harvested. Also, the results may not be applicable to field settings where roots have access to nitrate and ammonium, which is typically the case unless soil fertility is very poor. Total N uptake and fraction of N from symbiotic N fixation are both influenced by plant genotype and can vary independently (Dwivedi et al., 2015).

The costs and benefits of N fixation may vary over the course of plant development. During early plant growth, plants may benefit most by investing fixed N into leaves, thereby enhancing photosynthesis (Bethlenfalvay et al., 1978) and supporting all subsequent energy‐dependent processes, including N fixation. Later, during seed‐fill, plants may redirect the products of symbiotic N fixation toward seeds, thereby reducing the need to mobilize N from leaf rubisco into seed proteins (Sinclair & De Wit, 1976). To the extent that some strains induce plants to form nodules earlier, while others continue fixing N longer, early and late N fixation could be considered different traits. Therefore, comparing N fixation of different strains at a single developmental point may not predict season‐long benefits. To identify the most‐beneficial strain or mix of strains may require tracking N fixation throughout plant growth.

However, using total plant N content to track N fixation over growth (with or without isotope analysis) is typically destructive, so requires a large number of plants. Destructive harvests can also preclude comparing early and late data for the same plant. Combining measurements of N concentration and stable isotopes can provide a good proxy for N fixation and total N in whole plants, if combined with a nondestructive measurement of plant size (Muller et al., 2021).

Nondestructive measurements of nitrogen‐fixation rate are possible but challenging. The acetylene‐reduction assay is sensitive, but can be inaccurate if plants are mechanically disturbed (Minchin et al., 1986). Another source of error is the “acetylene‐induced decline” in activity, triggered by the cessation of N fixation that occurs with saturating acetylene concentrations (Minchin et al., 1983). Subsaturating acetylene can avoid the decline in nitrogenase activity, but extrapolating from the subsaturating acetylene reduction rate to the full N‐fixation rate is not trivial (Denison et al., 1983). Production of hydrogen, a by‐product of N fixation, can be measured without triggering a decline in nitrogenase activity. However, calculating the full N‐fixation rate from hydrogen production rate requires a brief exposure to an N‐free atmosphere, which can trigger a similar decline in nitrogenase activity (Minchin et al., 1983). Consumption of hydrogen by soil microbes, including some symbiotic rhizobia, is also a potential concern. An exciting new method for estimating N fixation nondestructively uses rhizobia with a fluorescent reporter linked to a key N‐fixation gene, so that nodules fluoresce more when fixing more N (Mendoza‐Suárez et al., 2020). The extent to which fluorescence reflects the abundance of rhizobial cells per nodule versus N fixation per rhizobial cell is not yet known, however. Any instantaneous measurement of N fixation rate requires multiple measurements to capture changes over plant development.

### 2.4 Net benefits also depend on carbon costs

A strain that makes more nodules will not necessarily fix more N. Furthermore, a strain that fixes more N or provides more of some other benefit will not always be more beneficial to the plant. Symbiotic microbes depend on plants for carbon and the cost to plants of supporting the microbes could exceed the benefit the plant receives. This problem would be particularly severe for mutualists out in the rhizosphere supported by root exudates, because those exudates may mostly support nonbeneficial or even pathogenic microbes. Even specialized exudates targeted to a specific beneficial species or strain may be used by other microbes (Gardener & de Bruijn, 1998), perhaps due to horizontal gene transfer and subsequent selection. This problem is less severe when resources are delivered directly to microbes within‐plant tissues (rhizobia, mycorrhizal fungi, and endophytes), but costs to the plant can still exceed benefits. For example, beyond some point, increasing nodulation can decrease rather than increase yields (Song et al., 1995).

**FIGURE 1:**
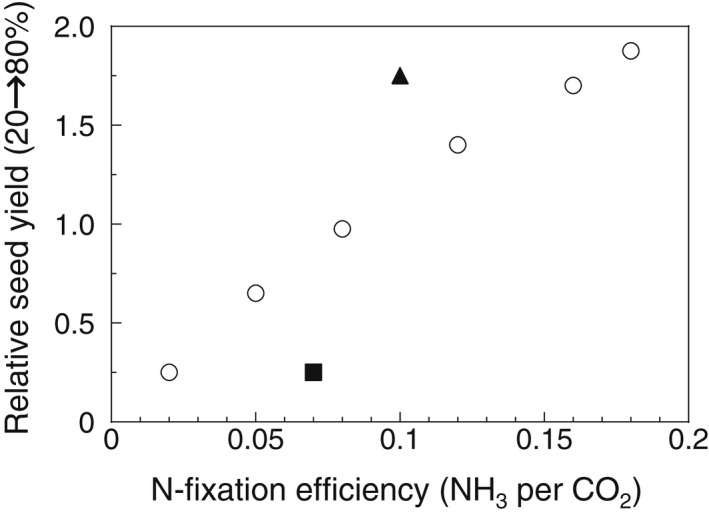
This graph of hypothetical data illustrates several points from the text. Nitrogen‐fixation efficiency is plotted, rather than rate per plant, to reflect the importance of carbon costs. Benefits are based on dry weight of seeds, because differences in early vegetative growth may not reflect final yield. Because complete replacement of less‐beneficial strains is impossible in the field, single‐strain inoculation may not predict real‐world performance. Instead, the vertical axis shows the effects of increasing the focal strain from 20% to 80% of the inoculum. Differences in nitrogen‐fixation efficiency explain differences in seed yield for most rhizobia strains (open circles), but one strain is more‐beneficial (triangle) and one strain less‐beneficial (square) than predicted from nitrogen‐fixation efficiency alone. These discrepancies could result from hormonal manipulation of the host plant, which could be either beneficial or harmful in a particular environment

Measurements of benefits from microbes should therefore be combined with measurements of costs, when possible, to compare the efficiency of different strains. For symbiotic N fixation, one measure of efficiency is the ratio of a strain's N‐fixation rate to its respiration cost (Figure 1). Both can be measured by gas exchange. However, only a fraction of nodulated‐root respiration is due to N fixation. A key insight was that small decreases in root‐zone oxygen concentration will decrease respiration in the oxygen‐limited nodule‐interior (directly linked to N fixation), with little effect on oxygen‐saturated respiration of roots or soil microbes (Witty et al., 1983). Efficiency can then be calculated from the marginal change in N fixation with a marginal change in nodulated‐root respiration. This approach has revealed significant differences in nodule‐operation efficiency between rhizobia strains and legume species. Efficiency increased both with host‐imposed changes in rhizobial bacteroids (Oono & Denison, 2010) and with knocking out rhizobial genes that divert carbon from N fixation to resource hoarding by rhizobia (Oono et al., 2020). Another possible measure of efficiency would combine the fluorescence assay for N fixation per nodule (Mendoza‐Suárez et al., 2020) with the same group's luminescence assay for C allocation to a nodule (Westhoek et al., 2021).

The efficiency of symbiotic N fixation also depends on the ratio of N fixed to nodule‐construction cost. Nodule mass per plant can now be estimated automatically from a photo of a nodulated root, using the artificial‐intelligence program, Root Painter (Denison, 2021; Smith et al., 2020). Measuring the ratio of shoot to root mass is even easier. In a comparison of plants inoculated with single rhizobia strains isolated from soils in Argentina, this root:shoot ratio was negatively correlated (r=0.94) with the soybean leaf concentration of ureides, thought to be a major transport form of N from nodules and therefore correlated with N fixation (Iturralde et al., 2019). It was suggested that plants reduced allocation to roots as N became less limiting, making shoot:root ratio a simple way to compare rhizobial strains, even with single‐strain inoculation (Iturralde et al., 2019). How widely this correlation applies even for nitrogen fixation is not yet known, but the same theoretical argument could perhaps be applied to mycorrhizae on P‐limited plants.

### 2.5 When might hormonal manipulation of crops by microbes be beneficial?

Many reported benefits from microbial symbionts involve some form of hormonal manipulation of the host plant. For example, in contrast to nitrogen supplied by rhizobia or phosphorus supplied by mycorrhizal fungi, any water‐related benefits from endophytes that are entirely enclosed within the plant cannot be due to the endophyte supplying water. These benefits have sometimes been attributed to “stomatal control” (Rho & Kim, 2017), which is presumably a hormonal effect. Some microbes do make plant‐hormone mimics (Mehmood et al., 2019), while others interfere with plant ethylene signaling (Ravanbakhsh et al., 2017). Both have been reported in rhizobia (Ma et al., 2002; Tatsukami & Ueda, 2016).

Because hormones are effective at such low concentrations, their metabolic cost to either plant or symbiont is probably negligible. Even defensive chemicals such as cyanogenic glucosides can have negligible biosynthesis costs, relative to their total effects on fitness (Kakes, 1989). Therefore, saving plants the metabolic cost of hormone production would not be a significant benefit. Instead, any benefits to the plant would depend on shifting plant resource allocation (e.g., between shoot and root) or other effects on plant phenotype (e.g., effects on stomatal opening). In such cases, the microbes would essentially be supplying information rather than resources (Denison, 2019).

Among well‐known symbionts, only mycorrhizal fungi extend out into the soil, potentially giving them access to current information not available to their plant hosts. For example, the common mycorrhizal networks that connect plants can transmit “infochemicals” (Barto et al., 2011) from pathogen‐infected potato plants, which trigger defenses in uninfected plants (Alaux et al., 2020).

Whenever information from one individual affects another, evolutionary biologists need to think about fitness effects on each individual. If supplying information is inadvertent (not under positive selection), we call that information a “cue” rather than a “signal” (van’t Padje et al., 2016). For example, it seems unlikely that a plant would benefit from giving competing neighbors advanced warning about pathogens, so a leaked chemical that provides that information would be considered a cue. From the mycorrhizal perspective, however, the same information‐bearing chemical might be considered a signal. This is because mycorrhizae benefit from providing hosts with information that keeps the plants healthy.

But collective benefits to mycorrhizae or other microbes are not necessarily enough to ensure that they will only transmit honest signals and only provide hormones that benefit plants. For example, a microbe that benefits from increased root growth nearby would be under selection to stimulate root growth, even if that growth consumed resources the plant would otherwise use to make seeds. To be maintained by natural selection, honest signaling to plants (or other forms of beneficial host manipulation) would need to provide individual microbes (or close relatives, including clones) with benefits not available to less‐beneficial strains on the same plant. This could depend on host‐imposed sanctions, discussed below.

This problem of individual versus collective benefits is not necessarily solved by vertical transmission of symbionts. Vertical transmission of symbionts in seeds gives symbionts a collective interest in the plant's reproduction, but multiple strains per plant can still create a tragedy of the commons. Which strain gets to go into the seeds and which has to stay behind to protect the leaves? Furthermore, hormonal manipulation that increases the production of seeds might not always be beneficial to plant long‐term fitness. For example, a seed‐transmitted symbiont might enhance its fitness by making a plant flower earlier than would be optimal for the plant's total lifetime fitness (Faeth, 2010) or for the total yield of perennial crops. Fungi that benefit more from vegetative or clonal growth of their hosts than from seed production can suppress sexual reproduction (Clay, 1991).

Phenotypic changes induced by microbial manipulation of plant hormonal systems also likely involve trade‐offs. For example, microbes that reduce ethylene production by plants can enhance growth in the absence of stress, but undermine stress tolerance (Ravanbakhsh et al., 2019). Similarly, a hormonally induced increase in root growth would consume resources that could have been used in other ways. This change in allocation might be beneficial under some conditions, but harmful under many others.

### 2.6 Measuring benefits or costs of manipulation from different strains

Quantifying any benefits to plants from manipulation by microbes is more difficult than for symbionts that provide resources such as nitrogen or phosphorus. Extrapolating from controlled environments to the field may be particularly prone to misleading results. If a plant species that evolved in the field is poorly adapted to a novel environment (pots in a greenhouse, say), then a phenotypic change in the right direction (e.g., increasing or decreasing root growth, depending on conditions) would be beneficial, so long as it was small enough to not overshoot the ideal phenotype. A random change would have a 50% chance of being in the right direction, which could result in an apparent benefit to the plant from manipulation of its phenotype by a symbiont, up to half of the time. But the same change might be harmful (in the wrong direction or too large) in a field environment to which the plant is better adapted.

Greater attention to mechanisms may be key to comparing benefits from different strains via manipulation of complex physiological functions. For example, do mycorrhizal fungi actually supply plants with water from the soil (leaving less water in the soil for later) or do they just manipulate plant transpiration? Greater “soil water extraction” was reported only five times in a three‐page table of mycorrhizal effects related to plant water (Auge, 2001). A recent meta‐analysis (Delavaux et al., 2017) identified putatively beneficial mycorrhizal effects on parameters of “water flow” (e.g., transpiration), but this was not true for parameters of “plant water content” defined to include water‐use efficiency. Water‐use efficiency (ratio of photosynthesis or yield to transpiration) is key to crop production in water‐limited environments. If benefits from manipulation are due to decreased transpiration, which would require either less leaf area or stomatal closure. Either of these would also decrease photosynthesis. Thus, beneficial effects on a plant's water potential could come with a cost of decreased photosynthesis by that plant, at least in the short term.

However, manipulation that imposes short‐term costs on individual plants might sometimes yield longer‐term benefits to the plant community and crop yield. A decrease in transpiration rate can conserve water in the soil to be used later, when it may be more beneficial. Some recently developed maize cultivars use this strategy. During the critical silking period, soil under two “drought‐tolerant” cultivars was actually wetter than under reference cultivars, because they used less water early (Cooper et al., 2014; Nemali et al., 2015). A similar short‐ versus long‐term trade‐off was seen after application of a transpiration‐suppressing film to drought‐stressed pea plants. Limiting transpiration decreased nitrogen‐fixation rate by symbiotic rhizobia for two days, but allowed greater N fixation on day 4 after application (Aldasoro et al., 2019). While these examples did not involve manipulation by microbes, these results are consistent with the possibility that microbial manipulation with short‐term costs to individual plants could sometimes benefit whole‐crop performance (Denison, 2019). Manipulation of host plants by microbes is the focus of another essay in this special issue (Klein et al., 2022).

### 2.7 Comparing strains offering multiple benefits is particularly difficult

A given microbial strain may provide more than one benefit to associated crop plants. For example, arbuscular mycorrhizal fungi (AMF) are best‐known for enhancing uptake of soil phosphorus, but they may also provide various other benefits such as access to other nutrients, abiotic stress tolerance, and defense against pathogens (Delavaux et al., 2017). When a species can provide multiple benefits, ranking strains from less‐ to more‐beneficial may depend on conditions. For example, to assess the relative importance of different benefits from mycorrhizal fungi, Sikes et al. (2010) applied structural equation modeling to data from two greenhouse experiments with pathogen‐exposed plants: one with *Plantago lanceolata* (Maherali & Klironomos, 2007) and one with *Setaria glauca* and *Allium cepa* (Sikes et al., 2009). The original experiment showed that mycorrhizal species differed in their effects on plant phosphorus (P) uptake and pathogen infection. The structural equation models showed that P uptake explained effects on plant biomass better than pathogen protection did. However, the relative influence of each benefit would presumably depend on environmental conditions, especially the degree of P limitation and the pathogen load.

In such cases, it can be difficult to interpret whether an apparent benefit arises directly from a particular activity of a microbial symbiont, or as a side effect of other benefits. For example, while numerous reports claim water‐related benefits from mycorrhizal infection, a mycorrhizal phosphorus benefit could change plant water status as a side effect. Plants with more access to P may develop a more‐vertical root architecture (Bonser et al., 1996), resulting in deeper rooting and more access to soil layers with more water.

The mechanism(s) through which symbionts benefit crops are important for several reasons. Fitness benefits and costs of the activity to the microbe, which are key to the activity's evolutionary persistence, will depend on its mechanism. So will potential trade‐offs, for both crops and microbes. The extent to which a microbial activity that is beneficial under some conditions is harmful under others will also depend on its mechanism. Ideally, therefore, we would like to measure each microbial process that has a benefit or cost to the plant. For example, how much nitrogen does a given strain of rhizobia fix and how much carbon does the plant expend in supporting that strain and its activities? If the strain hormonally manipulates plant water use, how are transpiration and water uptake affected? And how do each of these change over the growing season?

Unfortunately, however, it will often be impossible to be sure that we are measuring all benefits and costs, even in a particular environment. This is one reason to also measure net effects of different strains on plant growth. For example, if rhizobia strains differ in their net effect on plant growth in ways that cannot be explained by nitrogen fixation and its carbon costs, that suggests that rhizobia may be having additional effects, perhaps via hormonal manipulation (Figure 1).

### 2.8 Comparing net benefits of different strains using mixed inoculation

Measuring a strain's net effects on crop growth and yield may not be as simple as it seems, however. Under conditions where plants are infected only via inoculation—that is, not in the field—simply inoculating plants with one strain each and measuring plant growth is easy. But just comparing growth of plants inoculated with one mycorrhizal species or strain each will not necessarily predict which strain would be most useful in particular field conditions. What if one strain provides P even in cold soils, another is highly efficient (more P per C cost) later in the growing season, and a third protects from pathogens? Predicting the ideal mix from single‐strain experiments may be difficult or impossible. No matter how closely microclimate and nutrient supply match the target field environment—not very closely, usually—growth of a plant inoculated with a single strain is a risky proxy for that strain's contribution to yield in the field, where mixed infection is universal.

For example, comparing early vegetative growth of legumes inoculated with single strains of rhizobia may over‐emphasize rapid nodulation (Denison, 2021; Kiers et al., 2012). With single‐strain inoculation, strains with profuse early nodulation might support the most early vegetative growth, but this is a dubious proxy for final yield, if strains also differ in traits such as N‐per‐C efficiency or continuation of N fixation late in plant development. Fast‐nodulating strains could also appear to have greater fitness themselves, based on another dubious proxy: nodule occupancy in competition. Seed production and the final number of rhizobia in a plant's nodules are better proxies, respectively, for host and symbiont fitness in the field. Even so, single‐strain inoculation will miss synergistic effects from strains offering different benefits, including early versus late N fixation.

A better way to estimate a strain's potential contribution might be to compare plant growth or seed yield with different ratios (as few as two) of a focal strain, relative to either a reference strain or a mix (Friesen, 2012; Oono et al., 2009). A strain's potential contribution in real‐world mixtures might then be predicted by regressing yield on the percent of that strain in the inocula. However, strain interactions can be complex. For example, Heath and Tiffin (2007) reported that a mix of two rhizobia species resulted in less plant growth than the “worse” strain alone (albeit “worse” based on single‐strain inoculation). Antagonism between rhizobia strains (Schwinghamer, 1971), analogous to antagonism in the fungal gardens of ants (Poulsen & Boomsma, 2005), might undermine potential positive synergies. Antagonism among strains on root surfaces, competing for nodulation opportunities, is plausible, but might not reduce benefits to plants. If antagonism occurs within mixed nodules, however, it probably would reduce plant benefits.

In the field, an increase in the relative abundance of more‐beneficial strains (not complete displacement of less‐beneficial strains) is probably the most we can expect, either from improved inoculation or from increased plant‐imposed selection. The increase in crop yield with a strain's relative abundance is therefore the “gold standard” to identify more‐beneficial strains. For some symbioses, plant growth with single‐strain inoculation might be highly correlated with this standard, but we cannot simply assume this.

Comparing just two inoculation ratios might be sufficient to evaluate benefits of a strain. In Figure 1, the vertical axis is the ratio of seed yield for each focal strain when it is 80% of the inoculum, relative to 20%. However, using even two ratios doubles the number of plants needed, relative to single‐strain tests. A possible alternative or complementary approach is to use single‐strain inoculation, but measure efficiency parameters that differ little between single‐strain experiments and more‐realistic mixtures.

Ideally, we would measure both a symbiont's activities and resulting effects on crop variables and yield under production conditions. Often, however, symbiont activities (e.g., hormone production) are inferred rather than measured, plant measurements (e.g., growth rate) are not specific to those activities, and conditions in which measurements occur differ from production environments in ways that might affect results. For example, even if we only consider mechanisms based on hormonal manipulation, delayed wilting in a pot experiment could result from stimulation of root growth (increasing short‐term water uptake, but drying soil earlier) or reduced stomatal opening (conserving soil water for later use, but limiting photosynthesis). Distinguishing between these two mechanisms is a good first step, but predicting whether they would be beneficial in the field is more difficult.

We do not assume that plants have evolved optimal water‐related responses, even for conditions similar to where their ancestors evolved. However, plant phenotypes imposed by rhizobia or mycorrhizal fungi are probably even less likely to be consistently optimal for the plant. The microbes may have less information than the plant has about the relative merits of different plant phenotypes. Furthermore, the interests of fungus and plant are not identical, especially if multiple strains per plant create a potential tragedy of the commons, as discussed below.

## 3 HOW DOES A MICROBIAL STRAIN'S FITNESS DEPEND ON ITS NET BENEFITS TO CROPS?

This section focuses on the potential for crop‐imposed selection to favor more‐beneficial microbial symbionts. To maximize benefits to plants over years, more‐beneficial strains would need to reproduce more in symbiosis and/or survive longer in soil, relative to less‐beneficial strains, including less‐beneficial mutants of inoculum strains. In bacteria, the variation on which selection acts comes not from ordinary sexual recombination (half from each parent) but from various mechanisms, including mutation and horizontal gene transfer. For example, horizontal gene transfer from an inoculum strain helped indigenous rhizobia that were adapted to a wild legume host nodulate a newly introduced crop, on which they were less beneficial than the inoculum strain (Nandasena et al., 2007).

Argentina lacked soybean‐nodulating rhizobia before soybean was introduced, but inoculum strains applied today have to compete with the descendants of earlier inoculum strains, which had adapted to the environment. Iturralde et al. (2019) found that introduced soybean‐nodulating rhizobia in Argentina had evolved resistance to glyphosate, high temperatures, and the toxic forms of aluminum found in acid soils. It is not known whether horizontal gene transfer from indigenous bacteria was involved. Rhizobia traits that affect benefits and costs to plants have also presumably evolved.

### 3.1 Performance‐based, host‐imposed sanctions are needed to control “free‐riders”

The evolution of microbes that alternate between symbiosis and soil will be shaped by selection in both environments. Our central hypothesis, that interactions with plants have a major role in the evolution of microbial symbionts, depends on the relative importance of these two environments. Symbiont reproduction in association with a host may be many times greater than in soil. In a soybean field, for example, it was estimated from published data that more than 99% of rhizobia are inside nodules, not in the soil (West, Kiers, Simms, et al., 2002). A greenhouse study found that a labeled inoculum strain of rhizobia was about twenty‐fold more abundant in alfalfa rhizospheres than in rye rhizospheres (Miethling et al., 2000). When the soil had previously grown alfalfa, however, bulk‐soil populations of the inoculum strain were not significantly greater with alfalfa than with rye, perhaps because measurements were made prior to major release of rhizobia from nodules. Release of rhizobia from senescing soybean nodules can increase soil populations up to 200‐fold (Brockwell et al., 1987). Even a strain with only 7% nodule occupancy was ten times as abundant in the soil after soybeans were allowed to senesce, relative to where soybeans were removed prior to nodule senescence; a fivefold difference persisted for five years (Kuykendall, 1989).

If the evolutionary effects of microbe‐host interactions strongly influence which strains persist in soil, does symbiont evolution depend more on collective costs and benefits to symbionts or on the relative fitness of strains with host plants? West, Kiers, Simms, et al. (2002) modeled the effects of within‐host symbiont diversity on symbiont allocation to costly activities that benefit the host. With one strain per plant, strains that invested more in helping their host plant benefited from increased plant growth, relative to less‐beneficial strains infecting other plants. But, with realistic numbers of rhizobia strains per plant, the predicted fitness of strains investing anything in N fixation was less than that of “free‐rider” strains investing nothing.

Because this model prediction was inconsistent with the observed evolutionary persistence of legume‐rhizobia mutualism, they considered two alternative hypotheses, both involving host discrimination among root nodules differing in N fixation. Plants might reduce resource allocation to less‐beneficial nodules based on some fixed standard, or based on comparisons among nodules. Models based on either hypothesis predicted that substantial investment in N fixation by rhizobia would be evolutionarily stable (West, Kiers, Simms, et al., 2002). Such forms of discrimination have been called “host sanctions” (Denison, 2000). Although sanctions against rogue states may sometimes lead to better behavior, sanctions against microbes seem more likely to further reduce benefits they provide, by denying them resources. But individual plants imposing sanctions would benefit from using those resources for other purposes.

Consistent with these hypotheses, reduced allocation by legumes of resources to nonfixing root nodules appears to be universal (Chen & Thornton, 1940; Kiers et al., 2003; Oono et al., 2011), although this may not always reduce fitness of the rhizobia inside (Gubry‐Rangin et al., 2010). Host sanctions against less‐beneficial partners have also been reported in the mycorrhizal symbiosis (Kiers et al., 2011) and fig‐wasp mutualism (Jandér et al., 2016).

Even rhizobia that fix some N can be subjected to sanctions when a more‐beneficial strain is present (Quides et al., 2017), although we know less about such cases. This lack is partly due to the methodological challenges in measuring relative benefits from different N‐fixing strains, as discussed above. Also, host responses to N‐fixation rate or efficiency could be confounded by strain‐identity signaling or by hormonal manipulation of the host by the symbiont. This problem can be avoided by using a single strain and varying its N‐fixation rate using gas mixtures with different amounts of nitrogen. This approach revealed that one soybean cultivar imposed fitness‐reducing sanctions against rhizobia fixing N at 0 to 33% the rate of high‐fixation reference nodules on the same plant, whereas nodules fixing at 50% mostly escaped sanctions (Kiers et al., 2006).

Another way to avoid confounding performance‐based sanctions with effects of arbitrary identity signals or manipulation of hosts by symbionts is to use isogenic strains differing only in the actual benefit provided. Pea plants imposed sanctions on a rhizobia strain with about 25% the N‐fixation rate of an isogenic reference strain when the low‐fixing strain was coinoculated with a high‐fixing reference strain. However, the low‐fixing strain escaped sanctions when coinoculated with a nonfixing strain (Westhoek et al., 2021).

Some endophytes are transmitted vertically, in seeds. Vertical transmission can favor mutualism if there is only one symbiont strain per individual host plant, that is, no superinfection (Yamamura, 1993). This is because supporting reproduction of the host plant would tend to increase symbiont fitness, relative to symbionts on other plants. However, selection for symbiont mutualism might decrease with within‐plant symbiont diversity, just as it does without vertical transmission (West, Kiers, Simms, et al., 2002). Like *Dictyostelium* mixtures competing for a place in the spore (Strassman et al., 2004), multiple strains of endophyte within a host plant could compete for places in seeds in ways that might undermine total seed production. It is therefore not surprising that vertically transmitted endophytes in wild grasses can be parasitic (Faeth & Sullivan, 2003).

### 3.2 Less‐beneficial strains may be losers or cheaters

Some less‐beneficial symbionts are potential “cheaters,” i.e., strains that can benefit by diverting resources from host‐benefiting activities to their own reproduction, if they escape host sanctions. Some rhizobial cheaters may avoid sanctions by sharing nodules with more‐beneficial strains (Friesen & Mathias, 2010). However, some legumes impose sanctions on nonfixing strains even within mixed nodules (Daubech et al., 2017; Regus et al., 2017).

“Successful cheaters” may be defined as those that divert significant resources from N fixation to their own reproduction, without triggering sanctions severe enough to outweigh the rhizobia‐fitness benefit of resource diversion. For example, wild‐type rhizobia can accumulate the lipid, polyhydroxybutyrate (PHB) as 50% or more of their mass (Wong & Evans, 1971). It has been estimated that this much PHB could support the metabolism of sufficiently dormant rhizobia cells for years in soil between hosts (Muller & Denison, 2018). The cost to plants of PHB hoarding by rhizobia was first shown by the greater N accumulation of bean plants inoculated with a PHB‐minus knockout strain (Cevallos et al., 1996). This was confirmed by efficiency measurements; the knockout strain had a greater N‐fixation rate, relative to its respiration cost (Oono et al., 2020). Nodules containing the wild‐type strain were smaller than those with the more‐beneficial mutant, consistent with sanctions against PHB hoarders. However, the widespread evolutionary persistence of PHB hoarding suggests that its fitness benefits to rhizobia often exceed fitness risks of host sanctions.

Because PHB hoarding is so widespread, calling all PHB‐hoarders “successful cheaters” would make this a very abundant category. It has been suggested that cheaters “be identified by how their performance compares with other strains” (Kiers & Denison, 2008). A “free‐rider” strain that contributes less than other strains to the plant upon which they all depend is cheating those other strains. From the plant's perspective, a less‐beneficial rhizobia strain is harmful, even if it fixes enough N to justify its C cost, if it occupies a nodule that would otherwise be occupied by a more‐beneficial strain.

### 3.3 Cheating options are different for eusocial rhizobia

Some legume hosts impose terminal differentiation on rhizobia as the rhizobia differentiate into the N‐fixing, bacteroid form in nodules. This host trait, apparently based on plant peptides (Van de Velde et al., 2010), has evolved repeatedly (Oono et al., 2010), probably because it increases N‐fixation efficiency (Oono & Denison, 2010). Soil populations of rhizobia released from nodules of these hosts are descended from still‐reproductive rhizobia that have not yet differentiated into nitrogen‐fixing bacteroids. These rhizobia have been termed “eusocial” because of this extreme reproductive skew (Denison, 2021).

Sanctions on nonfixing strains within mixed nodules (Daubech et al., 2017; Regus et al., 2017) might have little evolutionary effect on rhizobia whose legume hosts prevent bacteroids from reproducing. These legumes might enhance their own fitness by preferentially allocating resources to more‐efficient bacteroids in mixed nodules. However, it seems unlikely that this would preferentially benefit the reproductive clonemates of those bacteroids (Denison, 2000).

PHB‐hoarding by nonreproductive bacteroids themselves is presumably rare (Oono et al., 2009), although we have not seen a comprehensive survey. However, some eusocial rhizobia have apparently evolved a novel cheating strategy in which bacteroids divert resources from N fixation to their still‐reproductive clonemates in the same nodule (Denison, 2000). This transfer uses complex molecules known as rhizopines, which plants are presumably unable to intercept. In a test of 332 rhizobia strains from hosts that suppress bacteroid reproduction, about 12% of strains could catabolize rhizopines (Wexler et al., 1995). The evolutionary persistence of rhizopine production suggests that it is beneficial to the rhizobia, as mutants that knock out such complex traits must arise frequently. But, given the possibility of horizontal gene transfer, why is the trait found in only 12% of strains? Is this resource diversion sufficiently costly to the legume host that it often triggers sanctions? If so, maybe rhizopine production only enhances rhizobia fitness if the rhizobia also have mechanisms to manipulate the host to avoid sanctions.

### 3.4 It will be difficult to enhance beneficial manipulation of hosts by symbionts

Manipulation of hosts by symbionts can be either harmful or beneficial to the hosts. Given the risk of sanctions, successful cheating by rhizobia may sometimes require hormonal manipulation of host plants. For example, some rhizobia produce rhizobitoxine, which results in less plant growth but greater PHB accumulation by the rhizobia (Ratcliff & Denison, 2009). By interfering with plant ethylene signaling, rhizobia that produce rhizobitoxine can also increase nodulation opportunities (Sugawara et al., 2006), possibly beyond what is optimum for the plant. A contrasting example is production of gibberellin by rhizobia inside nodules. This limits further nodulation (Tatsukami & Ueda, 2016), giving the gibberelin producers priority access to plant resources. In both examples, manipulation presumably benefits symbionts but harms their hosts. This form of manipulation has been termed “coercion” (Rowe et al., 2018).

However, we suggested in the previous section that there might be cases where manipulation of plant phenotypes by symbionts would benefit agriculture. Manipulation could perhaps enhance plant adaptation to a new environment. Or, it might benefit the whole‐crop plant community at some cost to individual‐plant competitiveness. For example, it might be possible to develop strains that would limit a plant's water use early in the growing season, saving water in the soil for use during critical periods. However, strains that reduce their host's competitiveness against neighbors would likely reduce their own fitness as well. Such strains would therefore be unlikely to persist, but perhaps inoculation with manipulating strains could provide a temporary benefit in some cases.

### 3.5 Persistence of more‐beneficial strains without sanctions?

Many less‐beneficial strains may be “losers” with defects that, even without sanctions, would reduce their own fitness as well as that of their hosts (Friesen, 2012). These defects could include poor adaptation to some hosts by strains that are more successful on other hosts. Fitness‐reducing defects would, by definition, keep each loser strain rare, not causing problems for agriculture. However, the overall abundance of loser strains would depend on mutation‐selection balance. Collectively, they could be a large fraction of less‐beneficial strains, but this has yet to be determined.

There may be other situations where strains that are more beneficial to crops could have consistently greater fitness, even without crop‐imposed selection, although such cases may be rare. Consider pathogen suppression by antibiotics produced by beneficial microbes on or near the roots. Should we expect natural selection to maintain or increase the effectiveness of these antibiotics against root pathogens? If a fungal pathogen is one of a microbe's main competitors for resources in the rhizosphere, then mutants producing a more‐effective antibiotic could have a selective advantage. On the other hand, if the pathogens are just one of many competitors in the rhizosphere, there may be only weak selection for suppressive chemicals specific to those pathogens. It might be worth exploring whether toxins made by rhizobia to kill competing rhizobia (Schwinghamer, 1971) or other competitors could provide some protection against root pathogens, although once rhizobia are inside nodules there might be little individual‐fitness benefit to suppressing pathogens in the soil.

## 4 COULD STRONGER CROP‐IMPOSED SELECTION FOR MICROBIAL MUTUALISM BENEFIT AGRICULTURE?

Lasting improvements to symbiosis will usually require host‐imposed selection for more‐beneficial strains. Postinfection selection based on actual performance (sanctions) seems more promising than selection (e.g., during nodulation) based on strain‐identity signals (partner choice). Although legumes do discriminate among rhizobia strains, they do not consistently favor the most‐beneficial strains. For example, five of six mutants that lost the ability to fix nitrogen in alfalfa nodules retained their competitiveness for nodulation (Amarger, 1981). However, improvements to partner‐choice selection may be possible and will be discussed first.

### 4.1 Evolution‐proof partner choice?

We could presumably develop highly beneficial inoculum strains with novel recognition signals, coupled with crops that only accept symbionts with those signals. But less‐beneficial mutants that enhance their own fitness at the expense of the host will often retain those signals. Similarly, we could develop crops whose roots exude novel toxins or resources, along with beneficial inoculum strains that resist those toxins or use those resources. Again, mutants that retain traits key to their own fitness would out‐compete those that prioritize host fitness. Horizontal gene transfer among symbionts (Nandasena et al., 2007) may spread even complex cheating traits.

A more‐sophisticated approach to “evolution‐proof” partner‐choice mechanisms may be worth exploring, however. Consider a rhizobia cell at some small distance from a root that is chemically signaling the availability of nodulation opportunities. Rhizobia have only a limited ability to move through soil, especially when the soil is dry (Aroney et al., 2021). For example, three strains tested were able to move at least 1 cm in three days in soil at a water potential of −1.5 MPa, but only one did so at −5 MPa (Issa et al., 1993). Diffusion of chemical signals and cues may also be limited in dry soils. But if a rhizobia cell is close enough to detect the root and near enough that it could perhaps reach it, is attempting to do so likely to increase its fitness? If a rhizobia cell manages to found a nodule, it will reproduce a millionfold or more. But if rhizobia populations in soil are approximately stable, then this potential benefit must be balanced by a very low average chance of successfully founding a nodule. Furthermore, the rhizosphere may be more dangerous for rhizobia than bulk soil is, due to greater numbers of predatory protozoa (Ramirez & Alexander, 1980). If so, then rhizobia populations must have been selected over recent millennia for their ability to balance predation risk against the potential fitness benefit of founding a nodule, using chemical cues. Those cues include: (1) any cues inadvertently released by protozoa, (2) the strength of the plant's nodulation‐opportunity cues and signals—a stronger signal suggests that the root is closer, increasing the chance of reaching the root before competing rhizobia, and (3) quorum‐sensing signals (or cues) that predict the abundance of competing rhizobia nearby (González & Marketon, 2003). For example, when rhizobia populations are very high, quorum sensing can actually reduce nodulation rates (Jitacksorn & Sadowsky, 2008). Rhizobia may interpret high concentrations of quorum‐sensing cues as predicting a small chance of founding a nodule, relative to the greater risk of predation near a root.

How might we increase nodule occupancy by a more‐beneficial inoculum strain, by manipulating these cues? If a legume crop were bred or engineered to release false quorum‐sensing cues (Sanchez‐Contreras et al., 2007), which could reduce its attraction for wild‐type rhizobia. Crop plants that also release false predation cues could enhance this effect. Inoculum strains would then be selected or engineered to ignore these cues, giving them a competitive advantage for nodulation. Of course, these cues could also be ignored by some mutants of indigenous strains and perhaps by less‐beneficial mutants of the inoculum strain. However, ignoring the predation cues, in particular, would only enhance fitness during a brief window when nodulation opportunities are available. Strains that ignore predator‐proximity cues would be more subject to predation during their sojourn in the soil between hosts, so inoculum strains might need to be applied every time the legume is grown. Inoculum companies would probably not object to that limitation.

### 4.2 Could stronger host sanctions significantly improve symbiont pools?

An alternative or complementary approach would be to select or engineer crops to impose stronger selection on populations of potential symbionts for greater mutualism with host plants. For example, if soybean genotypes vary in sanctions, a genotype with stricter sanctions could enrich the soil with only the most‐beneficial rhizobia strains in its nodules.

Stricter sanctions may be an example of traits that could be beneficial in agriculture, despite having been rejected by past natural selection (Denison, 2012; Denison et al., 2003). Although future generations of legumes could benefit from a decrease in the abundance of mediocre rhizobia in soil, even mediocre rhizobia might provide an N‐starved individual plant with immediate individual‐fitness benefits exceeding their individual‐fitness cost to that plant (Denison, 2000). Therefore, ancestral sanctions traits that persist in today's crops may be weaker than would be optimal from a longer‐term and whole‐crop perspective.

It might seem that positive legacy effects from stricter sanctions (benefits to a plant's own seedlings) would enhance a plant's inclusive fitness. If so, then any “simple” genetic change (i.e., one arising frequently via mutation or recombination), which improves sanctions would have been favored by past kin selection, leaving less room for improvement by humans. However, potential beneficiaries of stricter sanctions would also include the competitors of a plant's seedlings. Competition among relatives can eliminate inclusive‐fitness benefits linked to the greater relatedness that results from limited dispersal (West et al., 2001).

Assuming that cultivars with stricter sanctions are identified, would they be genuinely useful? A cultivar that enriched the soil with the most‐beneficial, locally adapted rhizobia would tend to benefit subsequent crops. For that benefit to be significant, however, the number of rhizobia released from nodules would need to be large enough to change the composition of soil populations. As discussed above, this is likely, due to the large numbers of rhizobia released from nodules. But would the beneficial change in soil populations last long enough to significantly improve yield of the next soybean crop in a two‐year or longer rotation? Possibly. Rhizobia that nodulate soybean have persisted in soil for twenty years without their host (Narozna et al., 2015). However, if there are large trade‐offs between a strain's benefit to its host and its ability to survive in soil, then beneficial effects of stricter sanctions might disappear by the time soybeans were next grown.

Also, a stricter‐sanction crop might, by shutting down more of its nodules, suffer from N deficiency. This would mainly be a problem the first‐time stricter‐sanctions cultivars were grown. This is because, in subsequent years, fewer nodules would be occupied by less‐beneficial strains and so shut down. So stricter‐sanctions crops might need supplemental N fertilizer, but total fertilizer use could still decrease if these crops were only needed occasionally.

Would supplemental N fertilizer applied to an occasionally needed, stricter‐sanctions cultivar also have beneficial evolutionary effects on rhizobia, by raising the N‐fixation threshold for rhizobia to avoid sanctions? Some strains whose N‐per‐C cost benefit exceeds that of soil N uptake, when soil N is low, would become uneconomic for plants, when soil N is high. On the other hand, high soil N can decrease nodulation, so that more nodules (including some less‐efficient ones) might need to be retained later. In theory, these two factors could balance each other (West, Kiers, Pen, et al., 2002). Timing of N availability is likely to be important. Experiments have not shown a consistently strong effect of soil N on sanctions (Oono et al., 2020; Regus et al., 2014).

It is unlikely that the crop genotype that is most beneficial to future crops at the site would also have the greatest yield, however. Even in a hypothetical collection of crop genotypes differing only in sanctions, optimizing symbiotic benefit:cost ratios for year‐zero plants might not optimize benefits to future crops (Denison, 2000). Although shutting down nonfixing nodules would benefit both current and future plants, the optimal treatment of mediocre nodules (fixing some N, but less N per C than other nodules on the same plant) is a complex issue beyond the scope of this paper.

So, too, is the possibility that crop genotypes might differ significantly in the ability to maximize short‐term benefits from a given rhizobia strain. It seems unlikely that sophisticated adaptations such as the host‐imposed manipulation that suppresses rhizobia reproduction and increases nitrogen‐fixation efficiency in some species (Oono & Denison, 2010) would vary drastically within species. But, for many farmers, even less‐extreme host adaptations that maximize current‐year benefits from symbionts might be of more value than evolutionary effects, even if the latter could provide more benefits within one or two years.

Experiments with a wild legume found little variation in sanctions among genotypes (Wendlandt et al., 2018). However, it would be premature to assume that this is also true for crops that have been subject to a range of selection regimes differing in N availability and other characteristics. For example, a six‐cultivar comparison found differences between older and newer cultivars in how the presence of nonfixing rhizobia affected crop growth (Kiers et al., 2007).

If stricter‐sanctions genotypes do exist, how might they be identified? One approach would be to develop sets of bacterial or fungal strains differing only in net benefits to the host. For rhizobia, the recent development of a 25%‐fixation strain is a promising step in that direction (Westhoek et al., 2021). Alternatively, a range of nitrogen‐gas concentrations could be used (Kiers et al., 2006).

Either way, we would need an efficient method to detect differences in sanctions severity against different nodules on the same host plant. Differences in viable rhizobia per nodule would probably be the best proxy for differences in numbers of rhizobia released into the soil, although the latter might not always be an accurate predictor of relative numbers in the soil (or nodule occupancy) two or more years later. For example, rhizobia leaving nodules with high PHB levels may reproduce up to threefold without an external carbon source (Ratcliff et al., 2012), so plate counts of rhizobia per nodule would underestimate the eventual abundance of high‐PHB strains in the soil.

In soybean, at least, sanctions appear to involve decreased oxygen supply to the nodule interior (Kiers et al., 2003). This decrease can be detected by noninvasive spectrophotometry of leghemoglobin (Denison & Layzell, 1991). To estimate a plant's threshold for sanctions, a nodule could be exposed to a gradual decrease in nitrogen gas concentration while monitoring leghemoglobin oxygenation. This would not necessarily be easier than counting viable rhizobia per nodule, but would give faster results than plate counts and might require fewer replicates, because each nodule would be compared with itself. However, methods like these are linked to specific sanctions mechanisms and so would only be applicable to a subset of symbiont species. We do not know of a comparable method for screening mycorrhizal fungi, for example.

An easy, mechanism‐agnostic screening method would build on existing field‐plot experiments. In year zero, use a field where replicated plots of different genotypes (e.g., soybean) are already being grown by plant breeders comparing yield or other crop traits. Record the location of each replicate plot so that it can be located in subsequent years. Tillage operations would need to minimize horizontal movement of soil among plots. In the next year, year one, a single reference cultivar of a typical rotation crop (e.g., maize) would be grown. Measure growth of the rotation crop at each plot location. Ideally, yield would be measured. However, a drone could be used to screen for significant (i.e., consistent among replicates) effects of year‐zero host genotype on vegetative growth. Next, in year two, grow a single reference cultivar of the year‐zero crop species and repeat the yield or growth measurements. If any year‐zero (e.g., soybean) genotype has particularly strict performance‐based sanctions on rhizobia, that should increase the year‐two growth and yield of soybeans at each location where that genotype was grown in year zero. If none of the year‐zero genotypes had effects in years 1 or 2 that were consistently positive across replicate plots, then no additional screening of those genotypes is needed. Either there were no large differences in sanctions among soybean genotypes, or the effects of those differences on soil populations of rhizobia disappeared over two years. It might, however, be worth testing a wider range of year‐zero genotypes.

What if some soybean genotypes are consistently more beneficial to year‐one maize, instead of, or in addition to, benefits to year‐two soybeans? Benefits to maize are unlikely to result from effects on rhizobia, but soybeans can affect the population of mycorrhizae that benefit maize (Johnson et al., 1992). There could also be legacy effects on other microbial mutualists or on pathogens. Possible legacy effects unrelated to microbes include persistent root channels (Rasse & Smucker, 1998). Genotypes with positive legacy effects could be useful to plant breeding programs, whatever the mechanism. But if such genotypes are identified, research into mechanisms would probably be worthwhile.

We have previously argued that the greatest opportunities to improve agriculture are in areas where natural selection has been constrained by trade‐offs that could be more acceptable by agricultural criteria, such as the lower competitiveness of shorter, higher‐yielding rice and wheat (Denison et al., 2003). Trade‐offs between individual fitness and community‐level benefits create such opportunities. So might trade‐offs between benefits to current versus future plant populations. The field experiment just proposed should provide some indication of opportunities and costs linked to these trade‐offs.

## 5 SUMMARY

- Microbial symbionts provide many benefits to crop plants, but significant improvements may be possible. We discussed methods that could be used to test this hypothesis, including ways to screen strains for greater mutualism and methods to identify crop genotypes that impose stronger selection for mutualism.
- Because plants in the field generally host multiple strains, relative benefits from different symbiont strains might best be assessed by regressing yield on a strain's abundance in an inoculum mixture. However, single‐strain experiments may be useful where mechanisms are understood and both costs and benefits are measured.
- “Host sanctions” that favor more‐beneficial strains (based on actual performance, not strain identity) have been documented in legume‐rhizobia and mycorrhizal symbioses.
- Crop genotypes with stricter sanctions could be identified in laboratory experiments or perhaps by planting a uniform test crop in a field that recently compared different genotypes of the crop. The latter approach might also identify other differences among genotypes in other legacy benefits they provide.

## CONFLICT OF INTEREST

None declared.

## Data Availability

N/A.
